# Seasonal trends and maternal characteristics as predictors of maternal undernutrition and low birthweight in Eastern Maharashtra, India

**DOI:** 10.1111/mcn.13087

**Published:** 2020-10-01

**Authors:** Lindsey M. Locks, Archana Patel, Elizabeth Katz, Elizabeth Simmons, Patricia Hibberd

**Affiliations:** ^1^ Department of Health Sciences, College of Health and Rehabilitation Sciences: Sargent College Boston University Boston Massachusetts USA; ^2^ Department of Global Health, School of Public Health Boston University Boston Massachusetts USA; ^3^ Lata Medical Research Foundation Nagpur India; ^4^ School of Medicine Boston University Boston Massachusetts USA

**Keywords:** anaemia, birthweight, body mass index, malnutrition, maternal nutrition, seasons

## Abstract

Few studies have assessed whether women and infants in rural and peri‐urban communities in South Asia experience seasonal fluctuations in nutritional status; however, a handful of studies have documented seasonal variability in risk factors for undernutrition including food availability, physical activity and infections. We used data from the Maternal and Newborn Health (MNH) registry, a population‐based pregnancy and birth registry in Eastern Maharashtra, India, to analyse seasonal trends in birthweight and maternal nutritional status—body mass index (BMI) and haemoglobin—in the first trimester of pregnancy. We plotted monthly and seasonal trends in birthweight, and maternal BMI and haemoglobin, and used multivariable regression models to identify seasonal and maternal characteristics that predicted each outcome. Between October 2014 and January 2018, MNH included 29,253 livebirths with recorded birthweight. BMI was assessed in 15,252 women less than 12 weeks of gestation and haemoglobin in 18,278 women less than 13 weeks of gestation. Maternal characteristics (age, education, parity and height) were significantly associated with nutritional status; however, there were minimal seasonal fluctuations in birthweight or maternal nutrition. There were significant secular trends in maternal haemoglobin; between 2014 and 2018, the prevalence of maternal anaemia decreased from 91% to 79% and moderate or severe anaemia from 53% to 37%. The prevalence of maternal underweight (45.3%) and overweight (9.8%) and low birthweight (19.1%) remained relatively constant over the study period. Our findings highlight that in some rural and peri‐urban areas in South Asia, tackling systemic drivers of malnutrition may be more effective than targeted interventions based on season.

**Key messages**

- Seasonal variability has been proposed as an important driver of undernutrition in the first 1000 days because of studies documenting seasonal fluctuations in risk factors including food availability, physical activity and infections.
- South Asia is considered particularly sensitive to seasonal trends because of the monsoon season.
- We did not find seasonal variability in the nutritional status of pregnant women or in birthweight in Eastern Maharashtra, India. Low birthweight and maternal undernutrition remained consistently high and were strongly correlated with maternal characteristics (age, education, parity and height).
- In Eastern Maharashtra, tackling underlying drivers of malnutrition may be more effective than targeting interventions based on seasonal risk factors.

## 1 INTRODUCTION

Globally, 15%–20% of births, or 20 million infants, are born with a low birthweight (LBW; <2500 g) (WHO, 2014). Southern Asia, with the highest prevalence of LBW worldwide, is home to an estimated 9.8 million LBW infants born each year (Blencowe et al., 2019). LBW infants have an increased risk of mortality, developmental delays and chronic diseases (Black et al., 2013). Undernutrition during pregnancy from conception through delivery—marked by low prepregnancy body mass index (BMI), inadequate gestational weight gain, micronutrient deficiencies and anaemia—are all major contributors to maternal and infant mortality and morbidity, including LBW (Black et al., 2013; Patel et al., 2018; Scholl, 2011; Victora et al., 2008).

In South Asia, the majority of undernourished women and children reside in rural areas where subsistence and semi‐subsistence agriculture serve as the primary livelihoods (Gillespie, van den Bold, Hodge, & Herforth, 2015; Kadiyala, Harris, Headey, Yosef, & Gillespie, 2014). Notably, agriculture is driven by annual cycles in weather, which affect labour demands, food supplies and disease vectors in the environment (Devereux, Sabates‐Wheeler, & Longhurst, 2013). In South Asia, the weather patterns are dominated by the monsoon season (which begins in June) followed by several months of cool weather (winter) before peak heat in April and May prior to the next monsoon cycle. For households living close to the poverty line, seasonal variations can have drastic negative health and economic consequences for individuals and communities (Devereux et al., 2013). Recently, seasonal variations have been proposed as an ‘indirect determinant’ of undernutrition in the first 1000 days in South Asia, particularly because of the effect of seasonality on agriculture and food systems (Madan, Haas, Menon, & Gillespie, 2018). If seasonal stress is a driver of malnutrition, this would warrant (1) further research into the specific drivers of seasonal variability and (2) an investment in programmes designed to address seasonal trends such as the use of supplementary feeding or social safety net programmes during lean seasons or programmes designed to target seasonal fluctuations in food systems, dietary intake, physical activity, infection and other individual drivers of malnutrition (Madan et al., 2018).

To date, season of birth has been shown to be associated with nutritional status in pregnancy, birthweight and offspring health in several studies in sub‐Saharan Africa (Anya, 2004; Moore et al., 1997; Prentice, Cole, Foord, Lamb, & Whitehead, 1987; Rayco‐Solon, Fulford, & Prentice, 2005; Rickard et al., 2012; Roba, O'Connor, Belachew, & O'Brien, 2015; Verhoeff, Brabin, Chimsuku, Kazembe, & Broadhead, 1999; Waterland et al., 2010). Fewer studies have assessed the effects of seasonality on the nutrition and health of women and children in South Asia, and no consistent seasonal trend has been identified (Bondevik, Lie, Ulstein, & Kvåle, 2000; Madan et al., 2018; Rao, Kanade, Yajnik, & Fall, 2009; Shaheen et al., 2006; Stevens et al., 2016). Research on the local impact of seasonality on nutritional status is essential for collecting high‐quality surveillance data as well as the development of context‐appropriate nutrition interventions. The discovery of wide seasonal variability in nutrition indicators indicates a need to carefully time surveillance, research or programme‐related surveys to account for seasonal trends; it would also warrant the need to conduct studies to further understand the specific local drivers of seasonal variability to strengthen local programmes and policies. By contrast, if there is no seasonal variability in nutritional status in a given area, this could highlight the importance of tackling long‐term, underlying drivers of poverty and malnutrition.

In this study, we utilize data from the ongoing Maternal and Newborn Health (MNH) registry in Nagpur, Eastern Maharashtra, India, to examine whether there were seasonal trends in birthweight and maternal nutritional status (BMI and haemoglobin concentration in the first trimester of pregnancy) in pregnancies from 2014 to 2018. Given the importance of poverty‐related risk factors for malnutrition, we also assess long‐term, nonseasonal, demographic characteristics as predictors of birthweight and maternal nutrition in this Central Indian population.

Mothers enrolled in MNH come from rural and peri‐urban households in the districts surrounding Nagpur City (Nagpur, Bhandara, Wardha and Chandrapur), where the majority of households work in agriculture. We thus hypothesized that there would be seasonal trends in maternal and child undernutrition given the seasonal fluctuation in important risk factors for undernutrition including food availability (Hillbruner & Egan, 2008; C Panter‐Brick, 1997), infectious diseases (Bondevik et al., 2000; Madan et al., 2018), agricultural workloads and energy expenditure (Panter‐Brick, 1993; Rao et al., 2009; Stevens et al., 2016). For women, we hypothesized that there would be an increased risk of malnutrition in women during the monsoon season and the months immediately after the monsoon, as has been documented in other studies in South Asia in different ecological zones (Bondevik et al., 2000; Hillbruner & Egan, 2008; Panter‐Brick, 1993). In most of South Asia, the monsoon season coincides with an increase in infectious diseases including malaria, influenza and dengue (Bondevik et al., 2000; Madan et al., 2018), increased agricultural workloads and energy expenditure (Panter‐Brick, 1993; Rao et al., 2009; Stevens et al., 2016) and lower dietary diversity (Hillbruner & Egan, 2008; Panter‐Brick, 1997). The one existing study of seasonality on birthweight in Maharashtra found that birthweights were highest in the summer (February–May), followed by the rainy season (June–September), and lowest in the winter (October–January) (Rao et al., 2009). Consistent with these findings, and given the importance of women's nutritional status in the second and third trimesters for birthweight (Hasan et al., 2018; Hickey, Cliver, McNeal, Hoffman, & Goldenberg, 1996; Siega‐Riz et al., 2009; Strauss & Dietz, 1999; Young et al., 2019), we hypothesized that children born in the winter (the season following the monsoon) would have the lowest birthweight as has been documented in two studies in South Asia (Rao et al., 2009; Shaheen et al., 2006). Although seasonality is likely to affect acute conditions that affect maternal and child nutrition, we also hypothesized that underlying maternal characteristics (age, education, parity and height) would influence our outcomes of interest and sought to quantify the relationship between these underlying characteristics and maternal and infant nutrition outcomes.

## 2 MATERIALS AND METHODS

### 2.1 Study population

This analysis uses data from the Nagpur, India, site of the Maternal and Newborn Health Registry (MNHR), a population‐based registry established in 2009 by the Global Network (GN) with support from *Eunice Kennedy Shriver* National Institute of Child Health and Human Development (NICHD), USA. The MNHR methods have been previously published (Goudar et al., 2012). The Nagpur site is composed of 20 clusters in four districts in Eastern Maharashtra (Nagpur, Bhandara, Chandrapur and Wardha). Each cluster is a geographic area surrounding a government primary health centre (PHC). MNHR aims to enrol all pregnant women residing in the geographic area as early as possible during pregnancy and then to follow the mothers and infants until 6 week post‐partum. Although the registry has been ongoing since 2009, beginning in late 2014, MNHR implemented changes to increase the proportion of women enrolled in MNHR in their first trimester of pregnancy. Given the change in recruitment protocol, we limit our analyses to women who enrolled between October 1, 2014 (when the expanded efforts for early identification of pregnant women began), and January 31, 2018 (the latest month with complete data available at the time of analysis).

At the first antenatal visit, information regarding the enrolled women's characteristics including age, education, parity, gestational age, height, weight and haemoglobin were recorded by a trained research administrator (a medical officer or auxiliary nurse–midwives employed at PHCs and subcentres). Weight was measured to nearest 100 g using a spring balance, and height to nearest centimetre was measured using a nonflexible measuring tape fixed to a wall. Haemoglobin was estimated within 2 weeks of enrolment using Sahli's method (Path, 1997). Birthweight was measured within 24 h of birth by using either a pan spring or a pan digital weighing scale. Data collection and management for the Nagpur site of MNHR have also been published previously (Patel et al., 2018). Briefly, trained registry administrators collected data on standardized paper forms. Each form was then manually reviewed prior to data entry at Lata Medical Research Foundation, Nagpur. After entry, data were transmitted to the GN data management centre (Research Triangle Institute, Durham, North Carolina, USA) where digital data checks were performed on a monthly basis. The data management centre forwarded edit, monitoring and performance reports to the Nagpur site team for data cleaning and management. The data were continually reviewed for quality by NICHD‐appointed data monitoring committee and by the Nagpur site data collection team.

### 2.2 Study sample and key variables

Our study sample is composed of all women enrolled in the MNH registry between October 1, 2014, and January 31, 2018. Mothers could enrol in MNH at any point in their pregnancy including before or immediately after labour and delivery. For our birthweight sample, we included all infants born between October 1, 2014, and January 31, 2018, with a recorded birthweight regardless of when the mother enrolled in the registry. Maternal nutritional status was assessed at the enrolment visit only, preventing an assessment of change in nutritional status (i.e. gestational weight gain) throughout pregnancy. Given that maternal BMI changes substantially over the course of pregnancy, to enhance comparability across mothers, we limited our maternal BMI sample to only mothers with BMI assessed in the first 12 weeks of pregnancy, which best approximates prepregnancy BMI. Maternal blood volume also changes dramatically over the course of pregnancy, resulting in different recommendations for anaemia cut‐offs based on pregnancy trimester (Centers for Disease Control and Prevention, 1989; WHO, 2011, 2016). We thus limited our maternal haemoglobin sample to only women who had their haemoglobin assessed in the first trimester of pregnancy (<13 weeks). All mothers who had their nutritional status assessed in the first trimester were thus eligible for inclusion in the BMI and haemoglobin samples. For the haemoglobin sample, haemoglobin assessment dates were confirmed to be within 13 weeks of the date of the reported last menstrual period. For the BMI sample, an additional survey question confirming that mothers had BMI assessed before 12 weeks of gestation was also used for inclusion criteria. Because the first trimester analyses aim to assess whether there are seasonal trends in maternal nutritional status in the first trimester, there were no exclusions for birth outcomes or subsequent maternal or neonatal morbidity or mortality. If a mother enrolled prior to January 31, 2018, and had her nutritional status assessed in the first trimester of pregnancy, she was eligible for inclusion in the BMI and haemoglobin samples, even if her child was born after January 31, 2018, and thus did not contribute to the birthweight sample.

Maternal first‐trimester BMI was calculated by dividing weight in kilograms (kg) by squared height in meters. Categorical variables were then created for the outcomes of interest. BMI classifications were based on the WHO expert consultation on BMI in Asian populations, which highlighted that although the international cut‐off for overweight is set at 25 kg/m^2^, in Asian populations, cardiometabolic risk increases with BMI > 23 (Barba et al., 2004). We defined underweight as BMI < 18.5 and overweight as BMI > 23 in accordance with the WHO consultation. Anaemia is defined as haemoglobin < 11 g/dl, moderate anaemia as 7–9.9 g/dl and severe anaemia as <7 g/dl in accordance with the WHO cut‐offs for women in their first trimester of pregnancy (WHO, 2011, 2016). Because severe anaemia was very rare in this population, moderate and severe anaemia were collapsed into a single category for analyses. LBW was defined as a birthweight < 2500 g. Cut‐offs for height categories (≤145, 145–149.9, 150–154.9 and ≥155 cm) were determined based on a meta‐analysis on the relationship of maternal short stature and offspring size for gestational age (Kozuki et al., 2015).

The Nagpur climate is classified as tropical wet and dry with a pronounced dry winter and heavy rain in the monsoon season (Geiger, 1954). Nagpur district experiences average annual rainfall of 1062 and 948 mm of which falls during the monsoon months from June to September (Observed Rainfall Variability and Changes over Maharashtra State, 2020). The prevalence of infectious diseases, including malaria and dengue fever, rises during the monsoon season and peaks in late monsoon and the months immediately after the monsoon (Dhiman, Shahi, & Sharma, 2005; Wasnik, Manohar, Humaney, & Salkar, 2012). The hottest month is May, with an average high of 42.4°C and an average low of 27.8°C. The coolest month is December with an average high of 28.1°C and an average low of 12.6°C. Seasonal data are analysed and presented based on calendar month and also by ‘seasons’. Cut‐offs for seasons were defined based on a review of the scientific literature from Nagpur and elsewhere in Eastern Maharashtra (Author, Tiple, & Khurad, 2009; Kakde, Kakde, & Saoji, 2001; Rao et al., 2009) combined with information from the U.S. national weather service. We defined summer as February–May, monsoon season as June–September and winter as October–January.

### 2.3 Data analysis

Descriptive statistics—frequencies with percentages and means with standard deviations—were used to present the socio‐demographic characteristics of the study samples. Data are presented for the birthweight, BMI and haemoglobin samples separately. Plots were created of the mean BMI, haemoglobin and birthweight as well as the prevalence of maternal underweight, overweight, anaemia, moderate or severe anaemia and LBW by month and season. Monthly and seasonal means and prevalences and their corresponding standard errors were obtained using survey procedures accounting for clustering. The seasonal data are presented in three ways: (1) by calendar month (averaged across years), (2) by calendar month and year and (3) by season (winter, monsoon or summer) and year.

To assess whether maternal characteristics and/or seasonal variables predicted birthweight, maternal BMI or maternal haemoglobin concentration, generalized estimating equations (GEEs), with an exchangeable correlation structure to account for clustering, was used. Underlying maternal characteristics of interest were selected based on a review of the literature and what was available in mothers in the MNHR. This included mother's age, education, parity and height. Although these characteristics are modifiable through long‐term poverty reduction and family planning interventions, they all precede the current pregnancy by years and are nonmodifiable in the context of ante‐natal care interventions aimed at improving nutrition during or immediately before pregnancy. Categories for predictors were used because the exposure–outcome relationships were assumed to be non‐linear. Univariable, reduced multivariable and full multivariable GEE models are presented. Reduced models were used to assess the relationship between maternal characteristics and each outcome of interest. All reduced models adjust for maternal age and education, and for the maternal BMI and maternal haemoglobin outcomes, the reduced models also adjust for gestational age at outcome assessment. Full multivariable models were used to assess the relationship between seasonality and each outcome for interest. The full multivariable models for birthweight adjust for maternal age, education, parity and height. For maternal BMI, the full multivariable models adjust for gestational age when weight and height were assessed as well as maternal age, education and parity, and for maternal haemoglobin, the full multivariable models adjust for gestational age when haemoglobin was assessed as well as maternal age, education, height and parity. The association between seasonality and each outcome was assessed with independent models defining seasonality in three different ways: (1) based on month of outcome assessment (January–December), (2) based on season of outcome assessment (winter, summer or monsoon) and (3) based on season with an interaction term for the year. Independent full multivariable models adjusting for underlying maternal characteristics (age, education, parity and height) were also used to assess maternal BMI and maternal haemoglobin in the first trimester as predictors of birthweight. Maternal nutritional status in the first trimester is excluded as a covariate in models assessing the association between seasonality and birthweight because these variables would be on the causal pathway through which season could affect birthweight.

### 2.4 Ethical approvals

The Maternal and Newborn Health Registry was approved by the Boston University Medical Campus and Lata Medical Research Foundation Institutional Review Boards. All mothers provided informed consent for themselves and their infants to participate in the study. The study was monitored by a Data and Safety Monitoring Committee appointed by NICHD. The committee reviewed the study annually.

## 3 RESULTS

Between October 1, 2014, and January 31, 2018, 35,387 women enrolled in the MNH registry in Nagpur, and there were 29,253 births with a recorded birthweight (our final sample for birthweight analyses). Infants whose mothers enrolled during this period but were born after January 31, 2018, were not included in birthweight analyses because their data were not available at the time of analysis, but their mothers were eligible for inclusion in the first‐trimester BMI and haemoglobin analyses; 19,092 women, 54% of all women who enrolled during this period, enrolled during their first trimester of pregnancy (<13 weeks of gestation). Among the women who enrolled in their first trimester, 18,278 women had a recorded value for haemoglobin before 13 weeks of gestation, and 15,252 has their weight and height assessed before 12 weeks of gestation.

Mothers who enrolled in the first trimester and comprised the BMI and haemoglobin samples had similar characteristics and infant birthweights to mothers in the birthweight sample (Table 1). The one exception was maternal BMI in the haemoglobin sample: Underweight women (BMI < 18.5 kg/m^2^) were more likely to have their haemoglobin assessed prior to 13 weeks gestation than women with a BMI ≥ 18.5 kg/m^2^. Fifty‐five percent of mothers in the haemoglobin sample were underweight compared with 46.2% and 45.4% in the birthweight and BMI samples. In all three groups, approximately half of women had a secondary education or less. Over three quarters of mothers were between 21 and 29 years of age, and half were pregnant for the first time. Maternal undernutrition—both chronic and acute—was highly prevalent in this population. Approximately two‐thirds of mothers were below 155 cm in height, and a quarter were below 150 cm. Over a third of mothers who had their BMI assessed in the first 12 weeks of pregnancy were underweight, and over eight‐tenths of those who had their haemoglobin assessed in the first 13 weeks of pregnancy were anaemic at enrolment.

**TABLE 1:** Characteristics of the study samples^a^

	**Birthweight sample** ***N* = 29,253**	**Maternal BMI sample** ***N* = 15,252**	**Maternal haemoglobin sample** ***N* = 18,278**
Maternal age			
≤20 years	3282 (11.2)	2019 (13.2)	2383 (13.0)
21–29 years	24,055 (82.2)	12,389 (81.2)	14,847 (81.2)
≥30 years	1916 (6.5)	844 (5.5)	1047 (5.7)
Missing	0 (0.0)	0 (0.0)	1 (0.0)
Maternal education			
Incomplete primary or secondary education (1–9 years)	4935 (16.9)	2231 (14.6)	2679 (14.7)
Secondary education (10 years)	10,233 (35.0)	5231 (34.3)	6274 (34.3)
More than secondary education (11+ years)	13,158 (45.0)	7491 (49.1)	8947 (48.9)
Missing	927 (3.2)	299 (2.0)	378 (2.1)
Parity			
No previous pregnancies	14,275 (48.8)	8082 (53.0)	9611 (52.6)
One previous pregnancy	11,963 (40.9)	5911 (38.8)	7129 (39.0)
≥2 previous pregnancies	2993 (10.2)	1252 (8.2)	1531 (8.4)
Missing	22 (0.1)	7 (0.0)	7 (0.0)
Maternal height			
<145 cm	1939 (6.6)	1026 (6.7)	1210 (6.6)
145–149.9 cm	5951 (20.3)	3078 (20.2)	3653 (20.0)
150–154.9 cm	11,970 (40.9)	6123 (40.1)	7396 (40.5)
≥150 cm	9391 (32.1)	5025 (32.9)	6019 (32.9)
Missing	2 (0.0)	0 (0.0)	0 (0.0)
Gestational age at enrolment			
<13 weeks	14,599 (49.9)	15,252 (100.0)	18,278 (100.0)
>13 weeks	14,654 (50.1)	0	0
Maternal BMI in the 1st trimester^b^			
<15 kg/m^2^	457 (4.0)	589 (3.9)	583 (3.2)
15–18.5 kg/m^2^	4848 (42.2)	6327 (41.5)	9463 (51.8)
18.5–23 kg/m^2^	5099 (44.4)	6839 (44.8)	6764 (37.0)
>23 kg/m^2^	1093 (9.5)	1497 (9.8)	1478 (8.0)
Not assessed in the 1st trimester^b^	17,756	0	3200
Mean BMI ± SE	19.2 ± 0.1	19.2 ± 0.1	19.2 ± 0.1
Maternal anaemia category^c^			
No anaemia (haemoglobin > 11 g/dl)	1633 (11.8)	2042 (13.5)	2399 (13.1)
Mild anaemia (8 g/dl ≤ haemoglobin < 11 g/dl)	5445 (39.3)	6122 (40.6)	7249 (39.7)
Moderate anaemia (7 g/dl ≤ haemoglobin < 8 g/dl)	6759 (48.8)	6896 (45.7)	8601 (47.1)
Severe anaemia (haemoglobin < 7 g/dl)	22 (0.2)	23 (0.2)	29 (0.2)
Not assessed in the 1st trimester^c^	15,394	169	0
Mean haemoglobin ± SE	9.9 ± 0.1	10.0 ± 0.1	10.0 ± 0.1
Birthweight			
<2500 g	5595 (19.1)	2394 (20.8)	2848 (20.6)
≥2500 g	23,658 (80.9)	9103 (79.1)	11,008 (79.4)
Missing^d^	0	3755	4422
Mean birthweight ± SE	2680 ± 11.5	2666 ± 10.5	2667 ± 10.4

^a^ All women who enrolled in MNH between October 2014 and January 2018 were eligible for inclusion. All infants with a recorded birthweight during this time period are included in the birthweight sample. Because weight and haemoglobin change substantially over the course of pregnancy, maternal BMI and haemoglobin analyses are restricted to women who completed assessments in the first trimester of pregnancy in order to ensure comparability of nutritional indicators. All women with haemoglobin assessed <13 weeks are included in the haemoglobin sample. A specific screening question with a dichotomous cut‐off for weight assessment at 12 weeks was used for BMI; thus, the maternal BMI sample includes only mothers whose weight was assessed before 12 weeks of gestation.

^b^ Proportion of women in each BMI category only provided among women who had BMI assessed in their first trimester of pregnancy.

^c^ Anaemia categories based on the WHO guidelines for defining anaemia in the first trimester of pregnancy (WHO, 2011, 2016). Severe anaemia is defined as hemogloin <7 g/dl, moderate anaemia as 7 g/dl ≤ haemoglobin <8 g/dl, mild anaemia as 8 g/dl ≤ haemoglobin <11 g/dl and no anaemia (haemoglobin > 11 g/dl).

^d^ Dataset does not include birthweight for births that occurred after January 2018.

### 3.1 Birthweight

**FIGURE 1:**
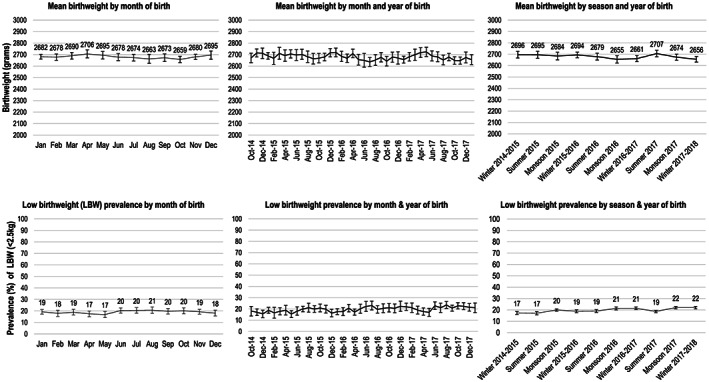
Monthly, annual and seasonal trends in birthweight among offspring of women who enrolled in the registry from October 2014 to January 2018 (*n* = 29,253)

Mean birthweight in our sample was 2681 g ± 446 (SD), with monthly means ranging from 2659 to 2706 g across the 12 calendar months (Figure 1). The overall prevalence of LBW was 19.1%, and it remained between 16.8% and 20.6% across the 12 calendar months. On average, birthweight was highest in the summer months (with a peak in April of 2706 g) and lowest during the monsoon and the months immediately following the monsoon (with a nadir in October of 2659 g). There was a small secular decline in mean birthweight from October 2014 to January 2018. The small differences in mean birthweight across calendar months and the secular decline were statistically significant in multivariable models adjusting for underlying maternal characteristics; however, the difference in means never exceeded 50 g (Table 2). There were, however, statistically and clinically significant differences in birthweight based on maternal characteristics. Maternal age, education, parity, height, BMI in the first trimester and anaemia status in the first trimester were all significantly associated with birthweight. Of particular note, in multivariable models adjusted for maternal age and education, short stature remained a significant predictor of birthweight: Mothers with statures below 145 cm gave birth to infants who were on average 206.2 g lighter than mothers with statures above 155 cm. In multivariable models adjusting for all underlying maternal characteristics (age, education, height and parity), maternal BMI in the first trimester of pregnancy was significantly correlated with birthweight: Mothers who were extremely underweight in the first trimester (BMI < 15 kg/m^2^) gave birth to infants who were 150.3 g lighter than mothers with BMIs in the ‘normal’ range (18.5–23.0 kg/m^2^).

**TABLE 2:** Predictors of birthweight in the Maternal and Newborn Health (MNH) registry in Eastern Maharashtra

	*n* *N* = 29,253	Birthweight (g) ± SE	Univariable		Reduced multivariable^a^		Full multivariable^b^	
			Diff. (95% CI)^c^	*P*	Diff. (95% CI)^c^	*P*	Diff. (95% CI)^c^	*p*
Underlying maternal characteristics								
Mother's age								
≤20 years	3282	2613 ± 13	−75.2 (−91.4, −59.0)	<0.001	−69.4 (−85.4, −53.4)	<0.001	‐	‐
21–29 years	24,055	2689 ± 11	Reference	‐	Reference	‐	‐	‐
>30 years	1916	2686 ± 20	−2.3 (−23.0, 18.4)	0.83	1.39 (−22.6, 25.4)	0.91	‐	‐
Mother's education								
<Secondary education (1–9 years)	4935	2646 ± 14	−65.7 (−80.2, −51.1)	<0.001	−73.6 (−86.9, 60.3)	<0.001	‐	‐
Secondary education (10 years)	10,233	2656 ± 14	−56.4 (−67.8, −44.9)	<0.001	−53.8 (−67.1, −40.5)	<0.001	‐	‐
>Secondary education (11+ years)	13,158	2712 ± 10	Reference	‐	Reference	‐	‐	‐
Missing	927	2676 ± 24	−36.1 (−85.7, −6.4)	0.02	−45.3 (−76.7, −13.9)	0.005	‐	‐
Parity								
No previous pregnancies	14,275	2638 ± 12	Reference	‐	Reference	‐	‐	‐
One previous pregnancy	11,963	2718 ± 12	79.9 (69.1, 90.7)	<0.001	85.9 (76.8, 94.9)	<0.001	‐	‐
≥2 previous pregnancies	2993	2726 ± 13	87.3 (69.9, 104.8)	<0.001	111.5 (92.0, 131.3)	<0.001	‐	‐
Missing	22	2730 ± 104						
Maternal height								
<145 cm	1939	2528 ± 14	−222.4 (−243.9, −200.8)	<0.001	−206.2 (−227.6, −184.8)	<0.001	‐	‐
140–149.9 cm	5951	2612 ± 11	−138.3 (−152.6, −124.0)	<0.001	−127.0 (−140.4, −113.6)	<0.001	‐	‐
150–154.9 cm	11,970	2683 ± 11	−67.1 (−79.0, −55.1)	<0.001	−59.1 (−70.3, −48.0)	<0.001	‐	‐
≥155 cm	9391	2750 ± 11	Reference	‐	Reference	‐	‐	‐
Missing	2	2350 ± 650	‐	‐	‐	‐	‐	‐
Maternal nutrition in the 1st trimester								
Body mass index (BMI)								
<15	457	2560 ± 24	−125.6 (−168.2, −83.1)	<0.001	‐	‐	−150.3 (−190.4, −110.1)	<0.001
15–18.5	4848	2.631 ± 10	−54.5 (−72.0, −37.1)	<0.001	‐	‐	−56.6 (−74.5, −36.6)	<0.001
18.5–23	5099	2686 ± 13	Reference	‐	‐	‐	Reference	‐
>23	1093	2769 ± 16	82.7 (53.7, 111.7)	<0.001	‐	‐	62.2 (26.3, 98.2)	<0.001
Not assessed in the 1st trimester	17,756	2689 ± 13	3.4 (−10.4, 17.3)	0.63	‐	‐	−16.9 (−37.6, 3.85)	0.11
Anaemia								
No anaemia	1633	2714 ± 20	Reference		‐	‐	Reference	
Mild anaemia	5445	2684 ± 10	−30.5 (−54.9, −6.0)	0.01	‐	‐	−26.0 (−49.8, −2.2)	0.03
Moderate or severe anaemia	6778	2643 ± 10	−71.4 (−95.4, −47.5)	<0.001	‐	‐	−37.1 (−67.0, −7.3)	−0.01
Not assessed in the 1st trimester	15,397	2691 ± 13	−23.0 (−45.5, −0.4)	0.046	‐	‐	−16.0 (−13.3, −42.1)	0.23
Month of birth								
January	2505	2681 ± 10	23.3 (0.4, 46.3)	0.046	‐	‐	23.6 (0.4, 46.8)	0.047
February	1833	2678 ± 13	19.8 (05.5, 45.0)	0.13	‐	‐	28.0 (7.9, 48.1)	0.006
March	2019	2690 ± 14	31.5 (7.0, 55.9)	0.01	‐	‐	38.5 (20.0, 59.4)	0.046
April	1981	2706 ± 17	47.8 (23.2, 72.4)	<0.001	‐	‐	53.1 (36.5, 69.6)	<0.001
May	2291	2694 ± 16	36.1 (12.6, 59.6)	0.003	‐	‐	42.4 (17.8, 67.0)	<0.001
June	2032	2678 ± 14	19.9 (−4.5, 44.3)	0.11	‐	‐	25.8 (3.5, 48.0)	0.02
July	2133	2674 ± 14	15.0 (−18.3, 27.7)	0.22	‐	‐	11.6 (−14.3, 37.4)	0.38
August	2469	2663 ± 19	4.7 (−18.3, 27.8)	0.69	‐	‐	2.9 (−19.3, 25.1)	0.80
September	2617	2673 ± 16	14.4 (−8.2, 37.1)	0.21	‐	‐	12.5 (−13.5, 38.5)	0.35
October	3447	2659 ± 13	Reference	‐	‐	‐	Reference	‐
November	3223	2680 ± 11	21.9 (0.5, 43.3)	0.04	‐	‐	16.8 (−1.3, 35.0)	0.07
December	2703	2695 ± 17	36.7 (14.3, 59.1)	0.001	‐	‐	31.2 (3.7, 58.7)	0.03
Season of birth								
Summer (February–May)	8124	2693 ± 13	20.9 (7.7, 34.2)	0.002	‐	‐	28.1 (15.2, 41.0)	<0.001
Monsoon (June–September)	9251	2671 ± 13	Reference	‐	‐	‐	Reference	‐
Winter (October–January)	11,878	2678 ± 11	6.1 (−6.1, 18.2)	0.33	‐	‐	4.0 (−6.1, 14.1)	0.43
Season & year of birth								
Summer 2015	2928	2696 ± 14	Reference	‐	‐	‐	Reference	‐
Monsoon 2015	2619	2695 ± 15	−0.6 (−24.1, 22.8)	0.96	‐	‐	5.8 (−11.6, 23.2)	0.51
Winter 2015–2016	3349	2684 ± 16	−11.7 (−33.8, 10.4)	0.30	‐	‐	−12.1 (−32.5, 8.2)	0.24
Summer 2016	3363	2694 ± 12	−2.5 (−24.5, 19.6)	0.83	‐	‐	−10.5 (−29.2, 8.2)	0.27
Monsoon 2016	3069	2679 ± 14	−16.7 (−39.3, 5.8)	0.14	‐	‐	−13.2 (−30.9, 4.5)	0.14
Winter 2016–2017	2965	2655 ± 16	−41.0 (−63.7, −18.3)	<0.001	‐	‐	−44.2 (−64.8, −23.6)	<0.001
Summer 2017	2803	2661 ± 13	−34.6 (−57.7, −11,6)	0.003	‐	‐	−40.5 (−55.1, −26.0)	<0.001
Monsoon 2017	2436	2707 ± 14	10.7 (−13.2, 34.6)	0.38	‐	‐	10.2 (−16.2, 36.7)	0.45
Winter 2017–2018	2937	2674 ± 13	−21.7 (−44.5, 1.1)	0.06	‐	‐	−30.0 (−9.0, −30.0)	0.01
Summer 2018	2784	2656 ± 12	−39.8 (−62.9, 16.7)	<0.001	‐	‐	−49.5 (−71.9, −27.0)	<0.001

^a^ Adjusted for maternal age and education only.

^b^ Adjusted for maternal age, education, parity and height.

^c^ Difference in means and corresponding 95% confidence intervals and *P* values obtained with generalized linear models accounting for cluster correlations using an exchangeable correlation structure.

### 3.2 Maternal BMI in the first trimester

**FIGURE 2:**
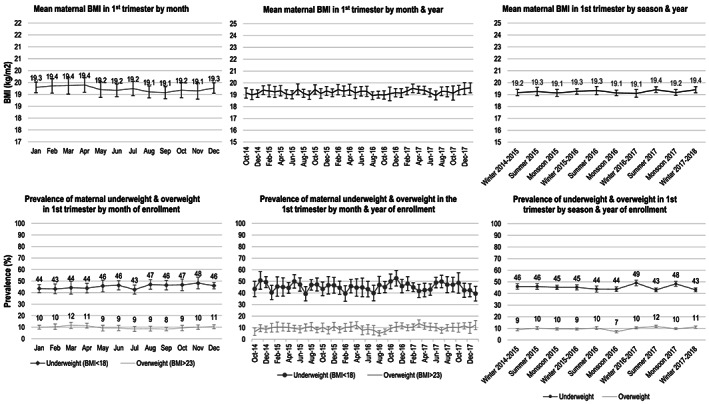
Monthly, annual and seasonal trends in maternal body mass index (BMI) among women who enrolled in the registry from October 2014 to January 2018 and were less than 12 weeks of gestation at the time of weight assessment (*n* = 15,252)

Mean BMI remained between 19.1 and 19. 4 kg/m^2^ across all 12 calendar months, with small month‐to‐month variability (Figure 2 **)**. The highest BMI means were observed in the summer months, peaking in February, March and April at 19.4 kg/m. Consistently, the prevalence of overweight was also highest in the summer months, with the peak of 11.6% in March, whereas the prevalence of underweight was highest after the monsoon season and during early winter, peaking in November at 48.3%. Of note, the overall prevalence of underweight (45.3%) and overweight (9.8%) remained relatively constant across calendar months ranging from 43% to 48% and 8%–12%, respectively, in individual months.

In multivariable models, maternal age, education, parity and month of enrolment were all significantly associated with BMI (Table S1). In the multivariable model adjusting for maternal education and gestational age at the time of weight and height assessment, maternal age remained strongly associated with BMI: Mothers over 30 years had mean BMIs that were on average 1.7 (1.3, 2.1) kg/m^2^ higher than mothers under 20 years. This corresponded with a notable difference in the prevalence of underweight (32.8% vs. 50.0%) and overweight (22.5% vs. 5.8%) among mothers over 30 years vs. mothers under 20 years. After adjusting for maternal age and gestational age at anthropometric assessment, educated mothers also had higher mean BMIs; mothers with more than a secondary education had mean BMIs that were on average 0.6 (0.5, 0.8) kg/m^2^ higher than mothers without a secondary education. Season of enrolment remained statistically significant in the multivariable models adjusting for maternal characteristics, but the differences were small. Mothers who enrolled in the summer months (February–May) had a mean BMI that was 0.2 (0.0, 0.3) kg/m^2^ higher (*P* = 0.01) than mothers who enrolled during the monsoon and its proceeding months (June–September).

### 3.3 Maternal haemoglobin in the first trimester

**FIGURE 3:**
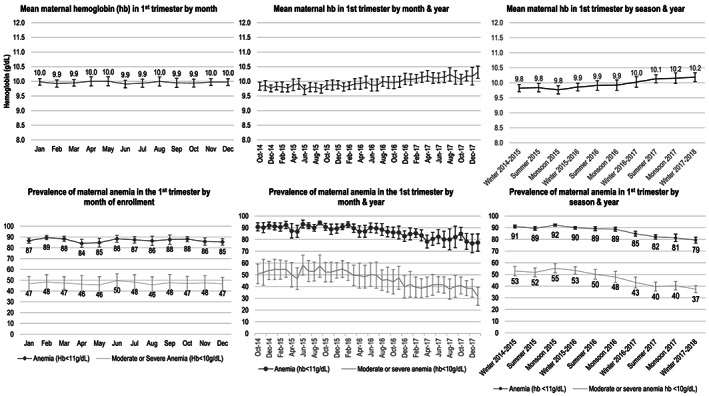
Monthly, annual and seasonal trends in maternal haemoglobin concentration among women who enrolled in the registry from October 2014 to January 2018 and were less than 13 weeks of gestation at the time of haemoglobin assessment (*n* = 18,278)

There was no clear seasonal trend in maternal haemoglobin in the first trimester of pregnancy; mean maternal haemoglobin remained between 9.9 and 10.0 g/dl in all 12 months of the calendar year **(**Figure 3 **)**. By contrast, there was a clear secular trend in maternal haemoglobin concentration with a significant increase over time from 2014 to 2018. Mean haemoglobin increased from 9.8 g/dl in winter of 2014–2015 to 10.2 in the winter of 2017–2018. The prevalence of anaemia decreased from 91% to 79% during this same time period and the prevalence of moderate or severe anaemia, which ranged from 50% to 55% in the early seasons of the study, decreased to 37% by winter 2017–2018.

Maternal characteristics were significantly associated with haemoglobin concentration (Table S2). In multivariable models, maternal age, parity and height were all significantly associated with haemoglobin concentration. Notably, mothers with more than a secondary education had an average mean haemoglobin concentration that was 0.32 (0.23, 0.42) g/dl higher than mothers who had less than a secondary education adjusting for maternal age and gestational age at time of haemoglobin assessment. After adjustment for gestational age at haemoglobin assessment, maternal age and maternal education, mothers with two or more previous pregnancies had haemoglobin concentrations that were 0.24 (0.18, 0.29) g/dl lower than primigravid mothers, and mothers with a very short stature (height < 145 cm) had concentrations that were on average 0.20 (0.13, 0.28) g/dl lower than mothers who were 155 cm or taller.

## 4 DISCUSSION

Our findings from this population‐based birth registry in Eastern Maharashtra indicate that there were no substantial seasonal fluctuations in birthweight or maternal nutritional status in the first trimester of pregnancy in this population. We did find, however, that underlying maternal characteristics (age, education, height and parity) were associated with significant differences in birthweight and maternal nutrition in the first trimester. Despite the limited seasonal variability, there were strong secular trends in maternal haemoglobin from 2014 to 2018, with the prevalence of anaemia and moderate or severe anaemia declining significantly during this time period.

The lack of notable seasonal fluctuations in maternal nutrition and birthweight in Eastern Maharashtra is surprising. The small seasonal fluctuations in maternal BMI (with peaks in February–April) may be a reflection of the fact that these ‘healthier’ months precede the lower food availability at the end of summer and increases in infection during and after the monsoon. However, the overall differences in BMI and the prevalence of underweight and overweight are relatively small and may not translate to clinical significance. Of note, these seasonal differences were not reflected in haemoglobin level or birthweight. Several studies of rural agricultural communities have documented strong seasonal trends in maternal and child nutrition, particularly in sub‐Saharan Africa (Anya, 2004; Bouvier et al., 1997; Chodick, Shira Flash, & Shalev, 2017; Hlimi, 2015; Hoestermann, Ogbaselassie, Wacker, & Bastert, 1996; Hounton et al., 2008; Moore et al., 1997; Prentice et al., 1987; Rayco‐Solon et al., 2005; Rickard et al., 2012; Roba et al., 2015; Verhoeff et al., 1999; Waterland et al., 2010). Research on the seasonality of maternal and child nutrition in South Asia is more limited. A recent review of 14 studies on seasonal variability and maternal and child nutrition in South Asia concluded that there is a compelling case that seasonal variation affects several of the proximal causes malnutrition in the first 1000 days—food access and availability, dietary intake, health and infection, maternal energy expenditure and child care practices (Madan et al., 2018). Notably, the review only identified four studies in pregnant and lactating women (Panter‐Brick, 1993; Rao et al., 2009; Schmid, Salomeyesudas, Satheesh, Hanley, & Kuhnlein, 2007; Stevens et al., 2016), with 10 additional studies in children, the majority of which assessed diarrhoea and infectious morbidity as their outcomes of interest. There may be greater seasonal variability in the risk factors for malnutrition in children than in pregnant women; however, this analysis does not include data on childhood malnutrition beyond birthweight. Individual studies in South Asia have found seasonal variability in determinants of nutritional status such as household food insecurity (Hillbruner & Egan, 2008; Stevens et al., 2016), dietary intake (Rao et al., 2009; Schmid et al., 2007; Stevens et al., 2016) and energy expenditure (Panter‐Brick, 1993; Rao et al., 2009); however, few studies include data on women's nutritional status or offspring birthweight as an outcome. One study from Nepal reported lower mean haematocrit and a higher prevalence of moderate or severe anaemia in the monsoon season (June through September) (Bondevik et al., 2000), and two separate studies (Rao et al., 2009; Shaheen et al., 2006), including one that was also conducted in Maharashtra (Rao et al., 2009), found a lower mean birthweight in the winter months. Notably, these studies were conducted in relatively small samples over short periods of time (1 year or less), sometimes using repeated cross‐sectional surveys that did not encompass a full year. In our large population‐based sample in Eastern Maharashtra, we enrolled women over the course of 40 consecutive months indicating that our findings of a lack of seasonal trends in the nutritional status of this population are not due to lack of representativeness or to timing of assessment. It is possible that other rural and peri‐urban communities in South Asia also do not experience seasonal trends in nutritional status and that there is a publication bias towards studies that do document seasonal variability.

Although our findings contradict the findings of other studies in South Asia documenting seasonal trends in nutrition and its determinants, our results do not undermine the importance of understanding of the specific seasonal trends, or lack of seasonal trends, in each setting prior to designing nutrition programmes and policies. The effect of seasonality on nutritional status depends upon the demographic characteristics and geography of each population studied. In this particular rural and peri‐urban population in Eastern Maharashtra, the prevalence of maternal undernutrition and LBW was consistently high across calendar months, highlighting the importance of nutrition interventions throughout the year in this population. Notably, maternal stunting and low education were robust predictors of nutritional status in pregnancy and birthweight in this population, indicating that long‐term poverty‐related risk factors may be more important drivers of nutrition in this study population of pregnant women than seasonal fluctuations in proximal determinants.

The alarmingly high rates of maternal anaemia in our study are similar to the findings of other studies conducted in the region (Ahankari, Myles, Dixit, Tata, & Fogarty, 2017; Corrêa et al., 2017; Patel et al., 2018). The 2015–2016 National Family Health Survey found that 50.7% of women aged 15–49 years living in rural areas in Nagpur district were anaemic (International Institute for Population Sciences, 2018), a discrepancy that may be due to the methods of haemoglobin collection. We previously reported virtually no change in the prevalence of anaemia in pregnant women in Nagpur from 2009 to 2016 in the general population but a decline in anaemia among women with a normal BMI (Patel et al., 2018). Our current analysis, which includes data up until 2018, indicates an observed decline in the prevalence of anaemia in the general population of pregnant women beginning in 2016, which may reflect the partial success of the Indian National Iron+ Initiative (Anand, Rahi, Sharma, & Ingle, 2014; Rai, Fawzi, Barik, & Chowdhury, 2018). Although India has had a National Nutritional Anaemia Prophylaxis Programme in place since 1970, the 2013 launch of the Ministry of Health and Family Welfare intervention guideline (Iron+) may have spurred the mobilization of greater resources for implementation in Eastern Maharashtra. The Iron+ Initiative aims to provide daily iron and folic acid (IFA) supplementation to pregnant and lactating women as well as weekly IFA supplements to women of reproductive age (15–49 years) through community health workers (ASHAs) and through schools for girls 10–19 years. Nationally, the coverage of IFA among pregnant women improved from 16% to 30% from 2006 to 2016 (Menon et al., 2019); this trend may have continued after 2016 with continued government investment. Notably, the observed reduction in the prevalence of anaemia between 2016 and 2018, without corresponding changes in underweight could be explained by an increased consumption of IFA supplements without major changes in other risk factors for undernutrition. Although the proportion of anaemia due to iron deficiency in this population is not known, globally, approximately half of anaemia is estimated to be due to iron deficiency (Stoltzfus, 2003), and the 2016–2018 Comprehensive National Nutrition Survey indicated that among adolescents in India (aged 10–19 years), 41.5% of anaemic adolescents (aged 10–19 years) also had iron deficiency. The attribution of anaemia due to iron deficiency may also be higher in Maharashtra where the prevalence of iron deficiency among 10–19 years old is 31.2%, higher than the national average of 21.5%. Despite the significant decline in the prevalence of anaemia in pregnant women in our study from 2014 to 2018, it should be noted that the prevalence of anaemia in pregnant women in Eastern Maharashtra remained above 75% throughout the study period, indicating that anaemia among pregnant women continues to be a severe public health problem in this population (WHO, 2011).

Our study has several limitations. Some mothers did not enrol in the MNH registry in their first trimester of pregnancy; thus, we do not have comparable maternal BMI or haemoglobin on all mothers; however, notably, there were no differences in key variables (birthweight or other maternal characteristics) between mothers in the birthweight sample and mothers with nutritional information from the first trimester of pregnancy. We also did not collect data on the change in nutritional status over the course of pregnancy, nor do we have data on dietary intake, energy expenditure or infectious diseases, so we are limited in our ability to assess seasonal variability in the determinants of nutritional status. The majority of households in Eastern Maharashtra are engaged in agriculture as their primary livelihood (Directorate of Economics and Statistics, Planning Department, G. of M., 2019); however, we did not collect individual‐level information on livelihoods and cannot identify which specific households engage in subsistence agriculture and thus could be more vulnerable to seasonal changes. Our study also has several strengths including the collection of birthweight data from a population‐based registry of 30,000 pregnancies over a continuous 40‐month period. Over half of pregnancies also included anthropometric and haemoglobin assessments in the first trimester of pregnancy. We also collected sociodemographic information about mothers, allowing us to adjust for important confounders. Our findings highlight that rates of maternal and child undernutrition in Eastern Maharashtra remain alarmingly high and that only maternal anaemia has begun to decline in recent years.

## 5 CONCLUSION

Research on the local impact of seasonality on nutritional status is essential for collecting high‐quality surveillance data as well as the development of context‐appropriate nutrition interventions. The discovery of wide seasonal variability in nutrition indicators indicates a need to (1) carefully time national or programme‐related survey assessments to account for seasonal trends and (2) conduct studies to further understand the specific local drivers of seasonal variability. Given the consistently high rates of undernutrition in Eastern Maharashtra, without seasonal fluctuation, and the equity implications of the strong correlation between undernutrition and underlying maternal characteristics, there is a clear need to strengthen large‐scale nutrition interventions in this population across all seasons.

## CONFLICTS OF INTEREST

The authors declare that they have no conflicts of interest.

## CONTRIBUTIONS

AP and PH designed the study and oversaw data collection; LML and ES analysed the data; LML, EK, PH and AP wrote the paper and had primary responsibility for final content. All authors read and approved the final manuscript.

## Supporting information


**Table S1:** Predictors of Maternal Body Mass Index (BMI) in the first 12 weeks of pregnancy
Supplemental Table 2: Predictors of maternal hemoglobin (hb) concentration in the first 13 weeks of pregnancy
