# Supplementary material for: Seasonal trends and maternal characteristics as predictors of maternal undernutrition and low birthweight in Eastern Maharashtra, India
Source: Matern Child Nutr. 2020 Oct 1;17(2):e13087. doi: 10.1111/mcn.13087 (PMC7988872; doi:10.1111/mcn.13087)
Supplement: Supplementary file 1 — Table S1: Predictors of Maternal Body Mass Index (BMI) in the first 12 weeks of pregnancy Supplemental Table 2: Predictors of maternal hemoglobin (hb) concentration in the first 13 weeks of pregnancy [file MCN-17-e13087-s001.docx]

| **Supplemental Table 1: Predictors of Maternal Body Mass Index (BMI) in the first 12 weeks of pregnancy** | | | | | | | | | |
| --- | --- | --- | --- | --- | --- | --- | --- | --- | --- |
|  | **N=15,252** | **BMI** ± **SE** | **Univariable** | | **Reduced multivariable^1^** | | | **Full multivariable^2^** | |
|  |  |  | **Diff. (95%CI)^3^** | **P** | **Diff. (95%CI)^3^** | **P** | **Diff. (95%CI)^3^** | | **P** |
| **Maternal characteristics** |  |  |  |  |  |  |  | |  |
| Gestational age (wks) at weight & height assessment | - | - | 0.0 (-0.0, 0.0) | 0.30 | 0.0 (-0.0, 0.0) | 0.16 | - | | - |
| Mother's Age |  |  |  |  |  |  |  | |  |
| ≤20 years | 2,019 | 18.8 ± 0.1 | reference | - | reference | - | - | | - |
| 21-29 years | 12,389 | 19.2 ± 0.1 | 0.4 (0.2, 0.5) | <0.001 | 0.3 (0.1, 0.5) | <0.001 | - | | - |
| >30 years | 844 | 20.5 ± 0.2 | 1.7 (1.4, 1.9) | <0.001 | 1.7 (1.3, 2.1) | <0.001 | - | | - |
| Mother's Education |  |  |  |  |  |  |  | |  |
| <Secondary education (1-9 yrs) | 2,231 | 19.0 ±.0 0.1 | -0.4 (-0.6, -0.3) | <0.001 | -0.6 (-0.8, -0.5) | <0.001 | - | | - |
| Secondary Education (10 years) | 5,231 | 19.1 ± 0.1 | -0.4 (-0.5, -0.3) | <0.001 | -0.4 (-0.5, -0.3) | <0.001 | - | | - |
| >Secondary Education (11+ years) | 7,491 | 19.4 ± 0.1 | reference | - | reference | - | - | | - |
| Missing | 299 | 19.4 ± 0.3 | -0.1 (-0.4, 0.3) | 0.69 | -0.2 (-0.7, 0.3) | 0.40 | - | | - |
| Parity |  |  |  |  |  |  |  | |  |
| no previous pregnancies | 8,082 | 19.1 ± 0.1 | reference | - | reference |  | - | | - |
| one previous pregnancy | 5,911 | 19.4 ± 0.1 | 0.2 (0.1, 0.3) | <0.001 | 0.2 (0.1, 0.3) | 0.003 | - | | - |
| ≥2 previous pregnancies | 1,252 | 19.3 ± 0.2 | 0.1 (-0.0, 0.3) | 0.12 | 0.0 (-0.2, 0.2) | 0.85 | - | | - |
| Missing | 7 | 21.2 ± 0.5 | - | - | - | - | - | | - |
| ***Month of Enrollment*** |  |  |  |  |  |  |  | |  |
| January | 1,477 | 19.3 ± 0.1 | 0.2 (-0.0, 0.4) | 0.051 | - | - | 0.1 (0.0, 0.3) | | 0.09 |
| February | 1,313 | 19.4 ± 0.1 | 0.3 (0.1, 0.5) | 0.02 | - | - | 0.3 (0.0, 0.5) | | 0.03 |
| March | 1,582 | 19.4 ± 0.2 | 0.3 (0.1, 0.5) | 0.01 | - | - | 0.3 (0.0, 0.6) | | 0.03 |
| April | 968 | 19.4 ± 0.2 | 0.3 (0.1, 0.6) | 0.001 | - | - | 0.3 (0.1, 0.5) | | 0.001 |
| May | 1,156 | 19.2 ± 0.1 | 0.1 (-0.1, 0.4) | 0.34 | - | - | 0.0 (-0.2, 0.2) | | 0.73 |
| June | 1,186 | 19.2 ± 0.1 | 0.1 (-0.1, 0.3) | 0.43 | - | - | 0.1 (-0.2, 0.3) | | 0.67 |
| July | 1,174 | 19.2 ± 0.1 | 0.2 (-0.1, 0.4) | 0.17 | - | - | 0.2 (0.0, 0.4) | | 0.06 |
| August | 1,352 | 19.1 ± 0.1 | 0.0 (-0.2, 0.3) | 0.81 | - | - | 0.1 (-0.1, 0.2) | | 0.58 |
| September | 1,256 | 19.1 ± 0.1 | reference |  | - | - | reference | | - |
| October | 1,342 | 19.2 ± 0.2 | 0.1 (-0.1, 0.3) | 0.37 | - | - | 0.1 (-0.1, 0.3) | | 0.35 |
| November | 1,380 | 19.1 ± 0.2 | 0.1 (-0.2, 0.3) | 0.55 | - | - | 0.0 (-0.2, 0.3) | | 0.86 |
| December | 1,409 | 19.3 ± 0.1 | 0.2 (-0.0, 0.4) | 0.10 | - | - | 0.1 (-0.1, 0.3) | | 0.22 |
| ***Season of Enrollment*** |  |  |  |  |  |  |  | |  |
| Summer (February-May) | 4,872 | 19.3 ± 0.1 | 0.2 (0.1, 0.3) | 0.001 | - | - | 0.2 (0.0, 0.3) | | 0.01 |
| Monsoon (June-September) | 4,772 | 19.1 ± 0.1 | reference | - | - | - | reference | | - |
| Winter (October-Jan) | 5,608 | 19.2 ± 0.1 | 0.1 (-0.0, 0.2) | 0.19 | - | - | 0.0 (-0.1, 0.1) | | 0.56 |
| ***Season & year of enrollment*** |  |  |  |  |  |  |  | |  |
| Winter 2014-2015 | 1,179 | 19.2 ± 0.1 | reference | - | - | - | reference | | - |
| Summer 2015 | 1,417 | 19.3 ± 0.2 | 0.1 (-0.1, 0.3) | 0.47 | - | - | 0.1 (-0.1, 0.3) | | 0.32 |
| Monsoon 2015 | 1,521 | 19.1 ± 0.1 | -0.0 (-.3, 0.2) | 0.78 | - | - | -0.0 (-0.2, 0.2) | | 0.80 |
| Winter 2015-2016 | 1,510 | 19.3 ± 0.1 | 0.1 (-0.1, 0.3) | 0.40 | - | - | 0.0 (-0.1, 0.2) | | 0.70 |
| Summer 2016 | 1,812 | 19.3 ± 0.2 | 0.2 (-0.0, 0.4) | 0.13 | - | - | 0.1 (-0.1, 0.3) | | 0.39 |
| Monsoon 2016 | 1,703 | 19.1 ± 0.1 | -0.0 (-0.3, 0.2) | 0.79 | - | - | -0.0 (-0.2, 0.1) | | 0.57 |
| Winter 2016-2017 | 1,697 | 19.1 ± 0.2 | -0.1 (-.03, 0.1) | 0.52 | - | - | -0.1 (-0.4, 0.1) | | 0.33 |
| Summer 2017 | 1,643 | 19.4 ± 0.1 | 0.2 (0.0, 0.5) | 0.03 | - | - | 0.2 (-0.0, 0.4) | | 0.05 |
| Monsoon 2017 | 1,548 | 19.2 ± 0.1 | 0.0 (-0.2, 0.2) | 0.98 | - | - | -0.0 (-0.2, 0.1) | | 0.61 |
| Winter 2017-2018 | 1,222 | 19.4 ± 0.1 | 0.2 (0.00, 0.5) | 0.046 | - | - | 0.1 (-0.1, 0.3) | | 0.27 |
| 1. Adjusted for gestational age at maternal weight and height assessment, maternal age and maternal education. 2. Adjusted for gestational age at maternal weight and height assessment, maternal age, maternal education and parity. 3. Relative risks and corresponding 95% confidence intervals and p-values were obtained with generalized linear models with the log link and binomial distribution accounting for correlations within clusters using an exchangeable correlation structure. | | | | | | | | | |

| **Supplemental Table 2: Predictors of maternal hemoglobin (hb) concentration in the first 13 weeks of pregnancy** | | | | | | | | | | |
| --- | --- | --- | --- | --- | --- | --- | --- | --- | --- | --- |
|  | **N=18,278** | **Hb ± SE** | **Univariable** | | **Reduced Multivariable^1^** | | | **Full Multivariable^2^** | | |
|  |  |  | **Diff. (95%CI)^3^** | **P** | **Diff. (95%CI)^3^** | **P** | **Diff. (95%CI)^3^** | | **P** |  |
| ***Maternal characteristics*** |  |  |  |  |  |  |  | |  |  |
| Gestational age at hb assessment (weeks)^3^ | 18,278 | - | -0.01 (-0.01, -0.00) | 0.01 | -0.01 (-0.02, -0.00) | 0.03 | - | | - |  |
| Mother's Age |  |  |  |  |  |  |  | |  |  |
| ≤20 years | 2383 | 9.9 ± 0.1 | -0.02 (-0.06, 0.02) | 0.25 | -0.00 (-0.06, 0.05) | 0.87 | - | | - |  |
| 21-29 years | 14,847 | 10.0 ± 0.1 | Reference | - | Reference | - | - | | - |  |
| >30 years | 1,047 | 10.0 ± 0.1 | 0.04 (-0.02, 0.09) | 0.21 | 0.04 (-0.04, 0.11) | 0.31 | - | | - |  |
| Missing | 1 | 10.2 ± 0.0 | - | - | - | - | - | | - |  |
| Mother's Education |  |  |  |  |  |  |  | |  |  |
| <Secondary education (1-9 yrs) | 2,679 | 9.8 ± 0.1 | reference | - | reference | - | - | | - |  |
| Secondary Education (10 years) | 6,274 | 9.9 ± 0.1 | 0.12 (0.08, 0.16) | <0.001 | 0.15 (0.09, 0.21) | <0.001 | - | | - |  |
| >Secondary Education (11+ years) | 8,947 | 10.1 ± 0.1 | 0.30 (0.26, 0.34) | <0.001 | 0.32 (0.23, 0.42) | <0.001 | - | | - |  |
| Missing | 378 | 9.8 ± 0.1 | -0.00 (-0.10, -0.10) | 0.98 | 0.01 (-0.12, 0.14) | 0.89 | - | | - |  |
| Parity |  |  |  |  |  |  |  | |  |  |
| no previous pregnancies | 9,611 | 10.0 ± 0.1 | reference | - | Reference |  | - | | - |  |
| one previous pregnancy | 7,129 | 9.9 ± 0.1 | -0.13 (-0.16, -0.10) | <0.001 | -0.11 (-0.15, -0.07) | <0.001 | - | | - |  |
| ≥2 previous pregnancies | 1,531 | 9.7 ± 0.1 | -0.30 (-0.35, -0.25) | <0.001 | -0.24 (-0.29, -0.18) | <0.001 | - | | - |  |
| Missing | 7 | 10.3 ± 0.2 | 0.28 (-0.40, 0.95) | 0.42 | - | - |  | |  |  |
| Maternal Height |  |  |  |  |  |  |  | |  |  |
| <145cm | 1,210 | 9.8 ± 0.1 | -0.30 (-0.36, -0.25) | <0.001 | -0.20 (-0.28, -0.13) | <0.001 | - | | - |  |
| 140-149.9cm | 3,653 | 9.9 ± 0.1 | -0.20 (-0.23, -0.16) | <0.001 | -0.12 (-0.16, -0.08) | <0.001 | - | | - |  |
| 150-154.9cm | 7,396 | 10.0 ± 0.1 | -0.12 (-0.15, -0.09) | <0.001 | -0.05 (-0.09, -0.00) | 0.04 | - | | - |  |
| ≥155cm | 6,019 | 10.1 ± 0.1 | Reference | - | reference | - | - | | - |  |
| ***Month of Enrollment*** |  |  |  |  |  |  |  | |  |  |
| January | 1,813 | 10.0 ± 0.1 | 0.07 (0.01, 0.14) | 0.02 | - | - | 0.07 (0.02, 0.12) | | 0.01 |  |
| February | 1,651 | 9.9 ± 0.1 | 0.02 (-0.05, 0.08) | 0.65 |  |  | 0.03 (-0.02, 0.07) | | 0.31 |  |
| March | 1,592 | 10.0 ± 0.1 | 0.04 (-0.03, 0.10) | 0.25 | - | - | 0.06 (-0.03, 0.15) | | 0.19 |  |
| April | 1,503 | 10.0 ± 0.1 | 0.09 (0.02, 0.16) | 0.01 | - | - | 0.10 (0.02, 0.17) | | 0.01 |  |
| May | 1,186 | 10.0 ± 0.1 | 0.09 (0.02, 0.17) | 0.01 | - | - | 0.10 (0.02, 0.17) | | 0.01 |  |
| June | 1,370 | 9.9 ± 0.1 | reference | - | - | - | reference | | - |  |
| July | 1,409 | 9.9 ± 0.1 | 0.03 (-0.04, 0.09) | 0.45 | - | - | 0.01 (-0.05, 0.07) | | 0.81 |  |
| August | 1,362 | 10.0 ± 0.1 | 0.09 (0.02, 0.15) | 0.01 | - | - | 0.05 (-0.03, 0.14) | | 0.20 |  |
| September | 1,453 | 10.0 ± 0.1 | 0.04 (-0.03, 0.11) | 0.22 | - | - | 0.00 (-0.08, 0.09) | | 0.98 |  |
| October | 1,606 | 9.9 ± 0.1 | 0.03 (-0.04, 0.10) | 0.38 | - | - | 0.01 (-0.05, 0.07) | | 0.64 |  |
| November | 1,609 | 10.0 ± 0.1 | 0.07 (0.00, 0.13) | 0.04 | - | - | 0.06 (-0.01, 0.13) | | 0.07 |  |
| December | 1,724 | 10.0 ± 0.1 | 0.07 (0.00, 0.13) | 0.049 |  |  | 0.05 (-0.01, 0.11) | | 0.09 |  |
| ***Season of Enrollment*** |  |  |  |  | - | - |  | |  |  |
| Summer (February-May) | 5,932 | 10.0 ± 0.1 | 0.02 (-0.02, 0.05) | 0.30 | - | - | 0.05 (0.00, 0.01) | | 0.03 |  |
| Monsoon (June-September) | 5,594 | 9.9 ± 0.1 | Reference | - | - | - | reference | | - |  |
| Winter (October-Jan) | 6,752 | 10.0 ± 0.1 | 0.02 (-0.01, 0.05) | 0.20 |  |  | 0.03 (-0.02, 0.08) | | 0.19 |  |
| ***Season & Year of Enrollment*** |  |  |  |  |  |  |  | |  |  |
| Winter 2014-2015 | 1,452 | 9.8 ± 0.1 | reference | - | - | - | reference | | - |  |
| Summer 2015 | 1,750 | 9.8 ± 0.1 | 0.02 (-0.05, 0.08) | 0.61 | - | - | 0.03 (-0.03, 0.09) | | 0.34 |  |
| Monsoon 2015 | 1,806 | 9.8 ± 0.1 | -0.06 (-0.12, 0.00) | 0.07 | - | - | -0.08 (-0.16, -0.01) | | 0.03 |  |
| Winter 2015-2016 | 1,906 | 9.9 ± 0.1 | 0.04 (-0.03, 0.15) | 0.26 | - | - | 0.01 (-0.09, 0.10) | | 0.89 |  |
| Summer 2016 | 2,160 | 9.9 ± 0.1 | 0.09 (0.03, 0.15) | 0.004 | - | - | 0.08 (-0.05, 0.20) | | 0.23 |  |
| Monsoon 2016 | 1,960 | 9.9 ± 0.1 | 0.10 (0.04, 0.16) | 0.001 | - | - | 0.08 (-0.05, 0.20) | | 0.26 |  |
| Winter 2016-2017 | 1,926 | 10.0 ± 0.1 | 0.20 (0.14, 0.26) | <0.001 | - | - | 0.17 (0.04, 0.29) | | 0.01 |  |
| Summer 2017 | 2,022 | 10.1 ± 0.1 | 0.31 (0.25, 0.37) | <0.001 | - | - | 0.27 (0.16, 0.38) | | <0.001 |  |
| Monsoon 2017 | 1,828 | 10.2 ± 0.1 | 0.33 (0.27, 0.40) | <0.001 | - | - | 0.25 (0.09, 0.40) | | 0.002 |  |
| Winter 2017-2018 | 1,468 | 10.2 ± 0.1 | 0.36 (-.30, 0.43) | <0.001 | - | - | 0.29 (0.12, 0.46) | | 0.001 |  |

1. Adjusted for gestational age at maternal hemoglobin assessment, maternal age and maternal education only
2. Adjusted for gestational age at maternal hemoglobin assessment, maternal age, education and parity.
3. Relative risks and corresponding 95% confidence intervals and p-values were obtained with generalized linear models with the log link and binomial distribution accounting for correlations within clusters using an exchangeable correlation structure
